# Using a Double-Sided Bronchial Blocker for Differential Lung Ventilation for Robotic Bronchoscopy-Guided Marking and Subsequent Bilateral Lung Wedge Resections and Lobectomy

**DOI:** 10.7759/cureus.70824

**Published:** 2024-10-04

**Authors:** Brett Dixon, Samuel Cohler, Jeffery DeGrauw, Ryan Hafen

**Affiliations:** 1 Kirk Kerkorian School of Medicine, University of Nevada, Las Vegas, Las Vegas, USA; 2 Anesthesiology, HCA Sunrise Health GME Consortium, Las Vegas, USA

**Keywords:** airway management, differential lung ventilation, double sided bronchial blocker, one-lung ventilation, robotic bronchoscopy, single-stage bilateral resection, thoracic surgery, wedge resection

## Abstract

One-lung ventilation presents unique challenges for the anesthesiologist. We present a case where the patient underwent robotic bronchoscopy to mark the lesion locations before bilateral wedge resections and a unilateral lobectomy. An 8.5 mm endotracheal tube was used to facilitate the robotic bronchoscopy. Subsequently, an EZ-Blocker double-sided bronchial blocker was placed to selectively isolate each lung during different phases of the procedure. This clinical situation required specific equipment, rather than a traditional double-lumen tube or a one-sided bronchial blocker, to efficiently manage the patient.

## Introduction

One-lung ventilation can pose unique challenges for the anesthesiologist. Successful one-lung ventilation techniques often require careful planning and a thorough understanding of the different uses of airway equipment. The decision to use a double-lumen tube versus a bronchial blocker is usually based on the preference of the surgeon or anesthesiologist; however, certain clinical situations may favor one over the other. When reliable separation of ventilation is needed, a double-lumen tube is often preferred [1]; however, as discussed in this case, a bronchial blocker may be the superior choice. 

An important consideration when using bronchial blockers involves ensuring the proper positioning relative to the right upper lobe bronchus. The distance between the start of the right main bronchus and the right upper lobe bronchus may be very short, potentially leading to a slow or incomplete collapse of the right upper lobe. This anatomical relationship increases the likelihood of bronchial blockers becoming dislodged [2]. On the other hand, double-lumen tubes have their own inherent drawbacks, such as difficulty in placement, as well as the risk of laryngeal injury and sore throat [3]. 

A significant advantage of using bronchial blockers is that the patient may remain intubated after surgery, whereas a double-lumen tube often needs to be exchanged for a traditional endotracheal tube [4]. While double-lumen tubes are more commonly used, understanding the clinical use of bronchial blockers is essential, as they may offer advantages in certain unique clinical situations. 

Written informed consent was obtained from the patient for the publication of this report. This report adheres to the case report (CARE) guidelines.

## Case presentation

**Figure 1:**
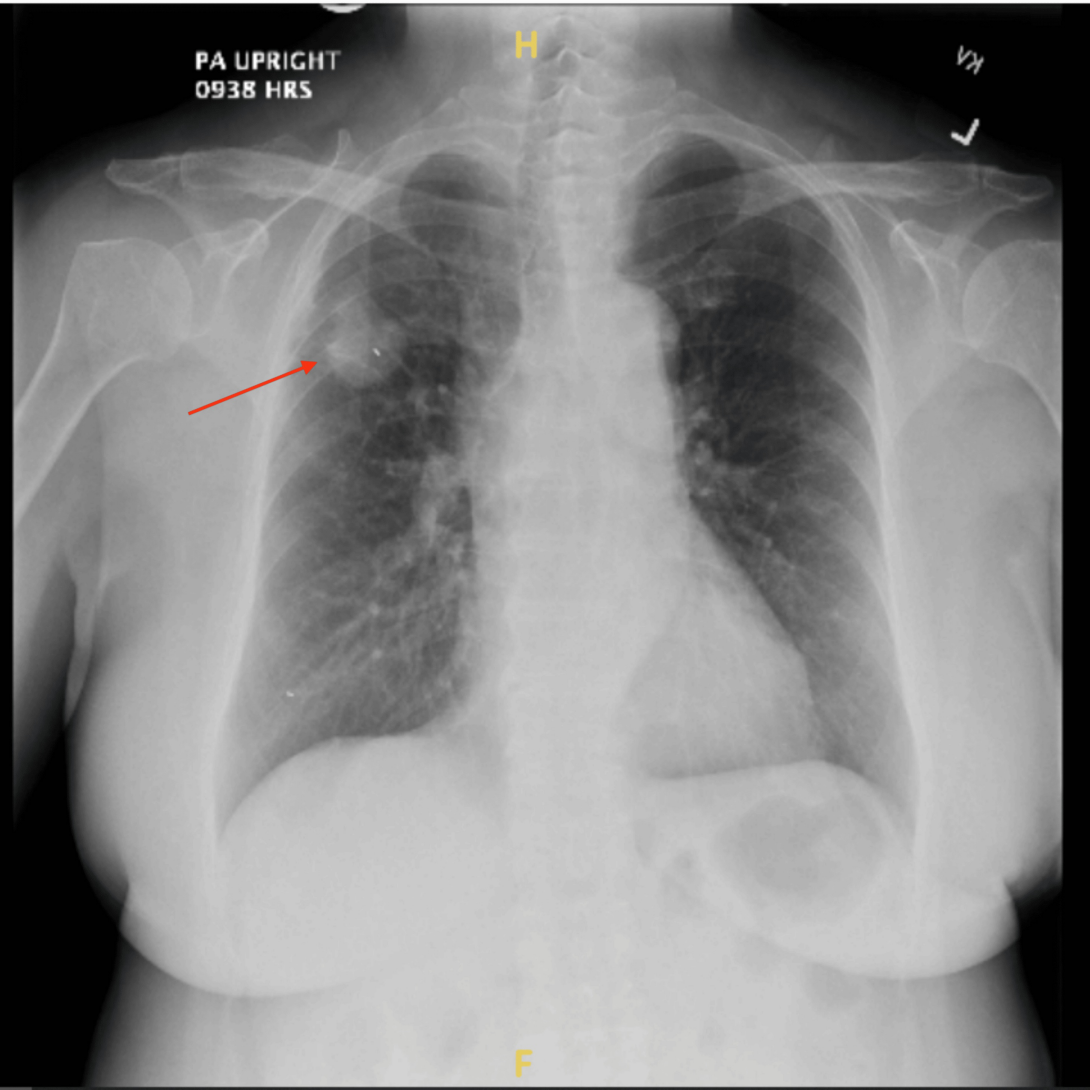
Right upper lobe mass identified on chest X-ray The arrow shows the right upper lobe mass. Ground glass nodules in the left and right lower lobes are better visualized on CT, which is unavailable CT: computed tomography

The patient was a 72-year-old female who presented for an elective robotic bronchoscopy with tumor localization, followed by potential bilateral pulmonary wedge resections. Her medical history included a right upper lobe mass and ground-glass nodules in the left and right lower lobes, identified on chest X-ray (Figure 1). She had never smoked and had no known allergies. Her vital signs were normal, with an oxygen saturation of 95% on room air, a temperature of 97.9 °F, a respiratory rate of 16 breaths per minute, blood pressure of 123/59 mmHg, and a pulse of 77 beats per minute. Over a 24-hour period, her intake was 1000 mL, and her output, including chest tube drainage and urine, totaled 1560 mL. The patient data are shown in Table 1.

**Table 1:** Patient data HR: heart rate

Parameter	Result	Reference range
Oxygen saturation (SpO_2_)	95%	95-100%
Temperature (Temp)	97.9 °F	97.8-99.1°F
Respiratory rate (RR)	16 breaths/min	12-20 breaths/min
Blood pressure (BP)	123/59 mmHg	90/60-120/80 mmHg
Pulse (HR)	77 beats/min	60-100 beats/min
Intake	1000 mL	Variable
Output	1560 mL	Variable

The physical examination revealed that the patient was alert and oriented. Her head and eyes were atraumatic, indicating no complications that could affect anesthesia or the surgical procedure. Her ears, nose, and throat (ENT) examination showed moist mucosal membranes, and she exhibited no respiratory distress, with symmetric chest expansion. Her extremities had a full range of motion, her neurological status was intact, and her psychiatric affect was normal. She denied experiencing fever, chills, or sweats, and her breathing was stable. 

Pathological examination confirmed adenocarcinoma in situ in both the left lower lobe and right lower lobe, and invasive acinar adenocarcinoma in the right upper lobe. The right upper lobe tumor measured 4.8 x 2.7 x 2.6 cm and was well-differentiated. There was evidence of visceral pleural invasion, and the bronchial margin was positive for adenocarcinoma. Metastatic carcinoma was found in one out of three regional lymph nodes.

Preoperative laboratory results and pulmonary function tests were within normal limits. Upon entry into the operating room, the patient’s identity was confirmed. Standard monitors were applied, and general anesthesia was induced with fentanyl, lidocaine, propofol, and rocuronium. An 8.5 endotracheal tube was used per the pulmonologist’s request to facilitate the robotic bronchoscopy. After induction, a left radial arterial line and a 16-gauge IV were placed for intraoperative monitoring. 

**Figure 2:**
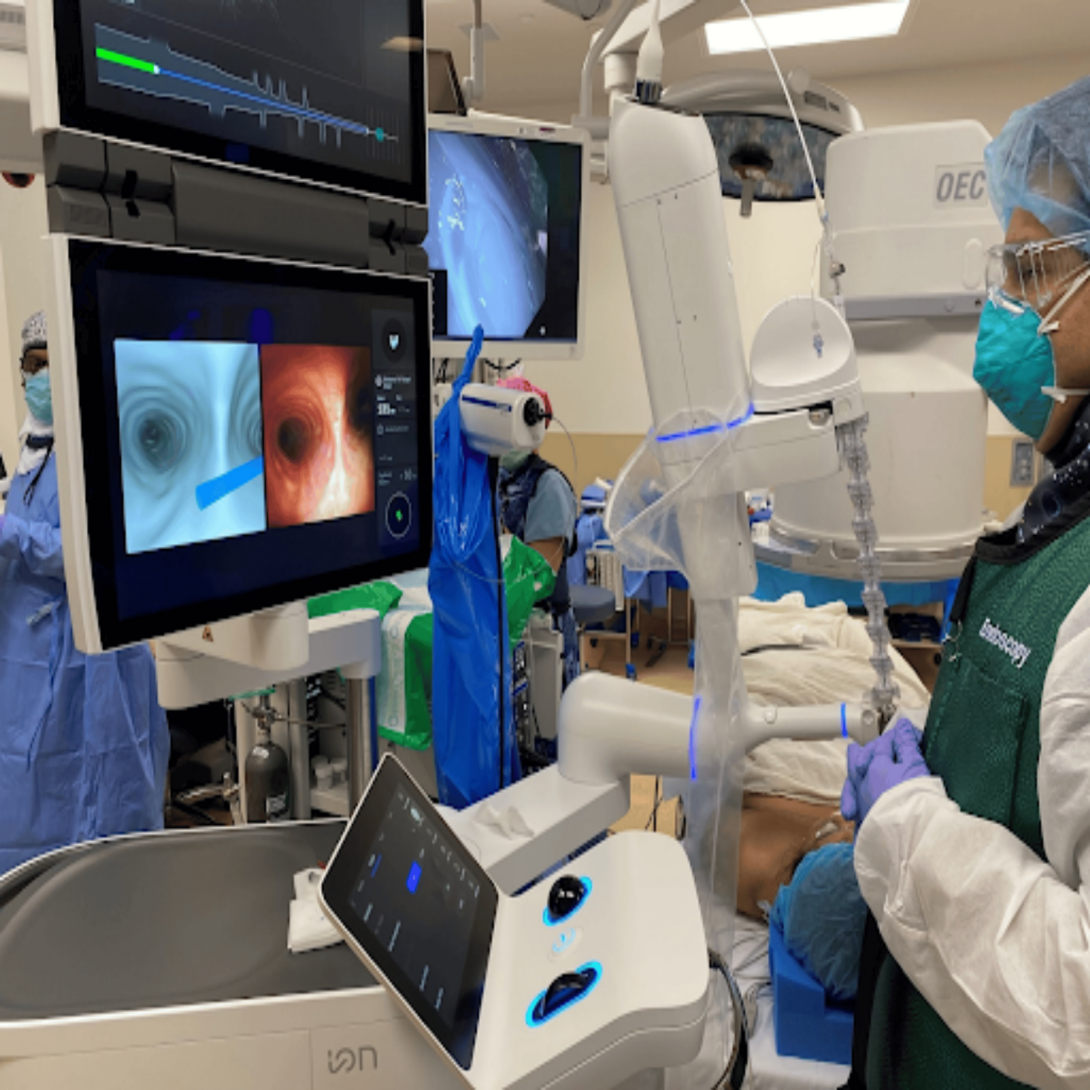
Robotic bronchoscopy to mark the locations of the nodules in the left lower lobe and the right lower lobe

**Figure 3:**
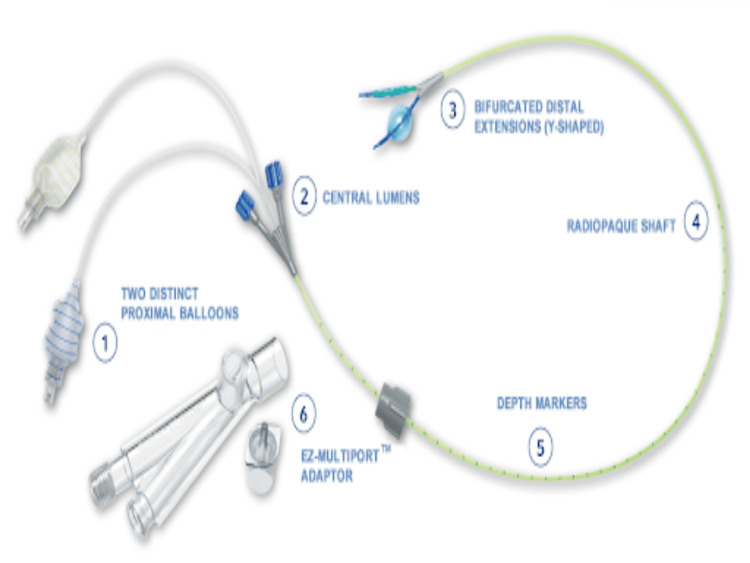
EZ-Blocker Endobronchial Blocker* *[5]

With the patient supine, the pulmonologist performed a robotic bronchoscopy, using dye to mark the locations of the nodules in the left lower lobe and the right lower lobe (Figure 2). Following the bronchoscopy, an EZ-Blocker double-sided bronchial blocker (Figure 3)** **was placed to isolate each lung, with proper placement confirmed via bronchoscopy. She was positioned in the right lateral decubitus position for the left lung procedure. The left lung was first isolated by inflating the left balloon of the bronchial blocker, which the surgeon confirmed. A wedge resection of the left lower lobe was performed and sent for frozen and gross pathological evaluation. After analyzing the frozen sample, the decision was made to proceed with the right lung. The left lung was re-inflated, and the patient was returned to the supine position before isolating the right lung. She was then placed in the left lateral decubitus position, the bronchial blocker was repositioned, and the right balloon was inflated. A right lower lobe wedge resection and a right upper lobectomy were performed. At the end of the procedure, normal ventilation was resumed, and an arterial blood gas analysis showed unremarkable results before extubation.

Following the surgery, the patient was admitted to the hospital, where she developed an air leak from her chest tube, likely related to the surgical procedure. Small air leaks are common after lung surgery and typically resolve over time, as was the case here. The patient was treated accordingly and discharged on postoperative day 12 following the removal of her chest tubes.

## Discussion

This case was selected for our report because it was the first case of a combined robotic bronchoscopy with bilateral thoracotomy performed in Nevada, highlighting the rarity of bilateral lung resections during the same anesthetic procedure. Over the past decade, numerous case reports and retrospective studies have shown the viability of single-stage bilateral lung resections. One study showed that single-stage bilateral pulmonary resection is a safe procedure, particularly when limited to wedge resection on at least one side [6]. Although patients who underwent single-stage bilateral pulmonary resection experienced longer mean operative time and duration of chest tube drainage, this study of 19 patients revealed no postoperative complications and no difference in postoperative hospital stay compared to those undergoing unilateral procedures. However, another study found that single-stage bilateral video-assisted thoracoscopic surgery (VATS) led to a higher postoperative leukocyte count and required longer postoperative ICU and hospital stays than the two-stage bilateral VATS approach [7]. Nonetheless, similar rates of postoperative complications, mechanical ventilation, and chest tube duration were observed between the two groups. Despite conflicting reports, there is a consensus that single-stage bilateral pulmonary resection is safe and effective provided patients undergo rigorous preoperative assessment [6-8].

Understanding the patient’s readiness for undergoing bilateral lung resection based on pulmonary function tests is crucial. Current guidelines use the predicted postoperative forced expiratory volume in one second (PPO FEV1) and the predicted postoperative diffusing capacity for carbon monoxide (PPO Dlco) to determine the appropriateness for surgery [7]. If both values exceed 60%, no additional tests are required. However, if either test result falls between 30 and 60%, an exercise test should be performed. If this test is negative, or if either value is less than 30%, a cardiopulmonary exercise test should be performed. If the cardiopulmonary exercise test shows a peak oxygen consumption of less than 35%, the patient is likely at high risk for mortality during the procedure. Given that this patient underwent resection of multiple lobes, it was important to calculate the patient’s PPO FEV1 and PPO Dlco from their preoperative pulmonary function tests to ensure that the procedure could be completed safely.

Besides evaluating the patient’s pulmonary function tests, the indications for performing a single-stage bilateral pulmonary resection over the traditional two-stage approach must be considered. Firstly, if there are concerns about the patient’s ability to tolerate multiple procedures, a single-stage approach would be favored. In the two-stage approach, the second operation is generally scheduled one to three months after the initial operation, but complications during this period may disrupt the postoperative course and delay further treatment. Second, for a single-stage approach to be feasible, suspicious nodules should be located peripherally on at least one side, enabling wedge or sublobar resections that preserve adequate pulmonary function [8]. Third, it is ideal that the patient is free of comorbidities that increase the risk of peri- or postoperative complications, such as diabetes mellitus, end-stage renal disease on dialysis, interstitial lung disease, or severe chronic obstructive pulmonary disease (COPD) [8]. 

Furthermore, Matsubara et al. proposed the following criteria for selecting patients to undergo a single-stage bilateral pulmonary resection for bilateral metastatic lung tumors: (1) the patient must be at low risk for pulmonary resection; (2) the primary lesion must be controlled, (3) no other metastatic disease (if present, it can be managed by surgery or other treatments, and modalities); and (4) all pulmonary lesions are resectable by wedge resection on at least one side [9].

Our patient fulfilled multiple criteria favoring single-stage bilateral pulmonary resection. The patient was a healthy 72-year-old female with no significant comorbidities such as diabetes mellitus, end-stage renal disease, or lung disease, aside from the bilateral lesions, which could increase the risk of peri- or postoperative complications. Preoperative laboratory results and pulmonary function tests were within normal limits, indicating the patient’s ability to tolerate bilateral pulmonary resection. According to Wang et al., this patient would be considered “low-risk” due to her health status and normal preoperative hemoglobin levels [10]. CT imaging and robotic bronchoscopy confirmed that the pulmonary lesions were located peripherally, allowing for wedge resection on one side. With no evidence of metastatic disease and the intention of curative treatment, the patient was a strong candidate for single-stage bilateral pulmonary resection.

Important anesthetic considerations included careful selection of appropriate airway equipment in consultation with the pulmonologist and surgeon. The airway equipment needed to facilitate bronchoscopy at the beginning of the case and allow for the isolation of either lung, depending on the stage of the thoracoscopic surgery. While double-lumen tubes are commonly used for thoracoscopic procedures and allow for lung isolation, they were not feasible in this case because robotic bronchoscopy would have been difficult through a double-lumen tube [11]. Additionally, the use of a double-sided bronchial blocker over traditional bronchial blockers made lung isolation more convenient. Unlike traditional bronchial blockers, which require repositioning to isolate the opposite lung, the double-sided bronchial blocker has a separate balloon for each bronchus, allowing for an easy transition between lungs during the procedure.

## Conclusions

In this case, a bronchial blocker proved superior to a double-lumen tube for a procedure involving robotic bronchoscopy and bilateral thoracotomy. The precise lung isolation provided by the bronchial blocker enabled a safe and efficient pulmonary resection. Comprehensive pulmonary function tests and CT imaging confirmed the patient’s suitability for a single-stage procedure, helping to avoid complications associated with the two-stage approach. 

This case underscores the importance of selecting optimal airway management techniques in complex thoracic surgeries to enhance patient outcomes. We emphasize the usefulness of a double-sided bronchial blocker in single-stage procedures, allowing for bronchoscopy at the beginning of the operation and subsequent independent lung isolation without the need to re-instrument the airway by placing a double-lumen tube. This approach reinforces the viability and safety of single-stage bilateral pulmonary resections, particularly in well-selected patients. Despite some conflicting evidence in the literature, the utility of single-stage procedures in such patients remains advantageous. The combination of robotic bronchoscopy with a double-sided bronchial blocker highlights advancements in airway management during thoracic surgery, offering valuable insights into optimizing procedures for complex pulmonary resections.
